# Society Meetings

**Published:** 1900-01

**Authors:** 


### SOCIETY MEETINGS.

The Niagara Falls Academy of Medicine held its regular monthly meeting at the office of Dr. C. O. Leo-Wolf, Silberberg Block, Monday, December 4, 1899, at 8.45 p. m. Dr. Leo-Wolf read a paper on Tubercular arthritis, its pathology, diagnosis and treatment. The discussion was opened by Dr. T. J. McBlain.

The Lake Erie Medical Society held its regular quarterly meeting at Dayton, N. Y., December 21, 1899.

The officers are: President, B. S. Bourne, Hamburg; vice-president, W. R. Henderson, Buffalo; secretary-treasurer, F. C. Beals, Salamanca; program committee, B. S. Bourne, Hamburg; F. C. Beals, Salamanca.

The following program was presented: Neurasthenia, W. J. French, Hamlet; Surgical knowledge of the prostate, E. J. Meyer, Buffalo; Valvular heart disease, H. C. Buswell, Buffalo; Thoughts on Bad-Nanheim, Germany, and its treatment of heart disease, M. B. Shaw, Eden.

The thirteenth International Medical Congress will be held at Paris, August 2-9, 1900. All doctors of medicine are entitled to membership in this congress by making a proper application and paying the sum of $5.00. The method of procedure is to fill out a blank, designate the section in which he desires enrollment, and send it with his visiting card and $5.00 to Dr. Henry Barton Jacobs, secretary, 3 West Franklin Street, Baltimore, Maryland. The secretary will forward it to Paris and the applicant will receive a certificate in due time. Rules and regulations governing the congress will be mailed upon application to the secretary. The committee hopes that the American representation in this important medical congress may be as large as possible and every member of the profession is urged to enter his name for membership, this alone entitling him to receive a digest of the full proceedings of the congress and a printed report of the section to which he may choose to attach himself.

---

THE Buffalo Academy of Medicine held meetings during the month of December, 1899, as follows:

*Section on Surgery.*—Tuesday evening, December 5th. Program: The value of normal salt solution in surgery with abstracts of cases, Chauncey P. Smith; Hypertrophy of the prostate, C. F. Durand; Dessicated suprarenal capsule in acute coryza, Frederick H. Millener.

*Section on Medicine.*—Tuesday evening, December 12th. Program: The relation of indicanuria to secretion of hydrochloric acid, Allen A. Jones. Personal experience with new drugs—General discussion.

*Section on Pathology.*—Tuesday evening, December 19th. Program: Meckel's diverticulum, with exhibition of specimens, Irving M. Snow; discussion opened by John Parmenter. Pathology of middle ear requiring radical treatment, George F. Cott; discussion opened by Max Keiser.

*Section on Obstetrics.*--Tuesday evening, December 26th. Program: Subject unannounced, Charles E. Congdon; The difficulties a young doctor encounters in obstetric practice, Nelson W. Wilson.

---

THE Medical Society of the State of New York will hold its ninety-fourth annual meeting at Albany, Tuesday, Wednesday and Thursday, January 30, 31 and February 1, 1900. The president, Dr. Willis G. Macdonald, of Albany has appointed the following business committee to whom all communications concerning papers to be presented at the next annual meeting should be sent. Dr. Wendell C. Phillips, 350 Madison Avenue, New York; Dr. Henry L. Elsner, Syracuse; Dr. Chauncy P. Biggs, Ithaca.

The seventy-ninth annual meeting of the Medical Society of the County of Erie will be held at the Y. M. C. A. building, corner of Mohawk and Pearl Streets, at 9 o'clock a. m., January 9, 1900. The following program has been prepared: Water supply and sewer problem of Buffalo, by the President, J. B. Coakley. The diagnosis of tumors of the breast—its importance, Chauncey P. Smith; Neuroses of childhood, Maud J. Frye; Unusual condition of the skin and kidneys following an operation for appendicitis, Wm. G. Taylor; Cardiac disease, Henry C. Buswell; Illegal medicine, Nelson W. Wilson.

This excellent list of papers would seem to justify the expectation of a full attendance, which the officers respectfully urge.

A change in the place of meeting should be noted and an earlier hour than usual has been named on account of the length and importance of the program.

---

The Buffalo Academy of Medicine has established a new section which embraces the study of diseases of the eye, ear, nose and throat. The organisation was perfected Monday evening, December 11, 1899, at which time officers were elected and a program arranged for the year. Dr. W. S. Renner was chosen as chairman, and Dr. R. H. Satterlee, as secretary. The meetings of the section will be held at the Academy parlors on the second Monday of each month, at 8 o'clock p. m. Dr. M. D. Mann, the president of the Academy, advocated the establishment of the section in a convincing speech, in which he said that it would tend to broaden the scope and increase the usefulness of the Academy.

---

The Southern Surgical and Gynecological Association held its twelfth annual meeting at New Orleans, December 5, 6 and 7, 1899, which was in all respects a conspicuous success. The president, Dr. Joseph Taber Johnson, of Washington, conducted the affairs of the association with great skill and dignity, the attendance was large and enthusiastic, the papers and discussions were able and of high order, the weather was beautiful, and everything seemed to conspire to make the occasion enjoyable and instructive.

The Orleans Parish Medical Society entertained the visitors at luncheon at the Boston Club on Tuesday, and provided an excursion down the Mississippi to a famous sugar plantation on Wednesday. The French Opera was open to the association Tuesday evening, where the Huguenots was most admirably sung, and the Tulane Theater kept open doors to members on Thursday evening.

It was altogether a rare treat to attend the meeting and will not soon be forgotten by those present.

Dr. Ernest S. Lewis, chairman of the committee, and his able lieutenants, Drs. W. E. Parker and Isidore Dyer, were everywhere in evidence and deserve great credit for the manner in which the association was entertained.

Officers were elected as follows: President, A. M. Cartledge, Louisville; vice-presidents, Manning Simmons, Charleston and W. P. Nicholson, Atlanta; secretary, W. E. B. Davis, Birmingham; treasurer, W. D. Haggard, Jr., Nashville. The next meeting will be held at Atlanta, beginning the second Tuesday in November, 1900.

